# Differences between children and adolescents who commit suicide and their peers: A psychological autopsy of suicide victims compared to accident victims and a community sample

**DOI:** 10.1186/1753-2000-6-1

**Published:** 2012-01-04

**Authors:** Anne Freuchen, Ellen Kjelsberg, Astri J Lundervold, Berit Grøholt

**Affiliations:** 1 Department of Psychiatry, Faculty of Medicine, University of Oslo, N-0316 Oslo, Norway; 2 Department of Child and Adolescent Mental Health, Sorlandet Hospital, N-4610 Kristiansand, Norway; 3 Centre for Forensic Psychiatry, Oslo University Hospital, N-0407 Oslo, Norway; 4 Department of Biological and Medical Psychology, Uni Research, K.G.Jebsen Centre for Reasearch on Neuropschyiatric Disorders, University of Bergen, N-5009 Bergen, Norway; 5 Institute of clinical medicine, Faculty of medicine, University of Oslo, N-0361 Oslo, Norway

**Keywords:** Children, adolescents, suicide, fatal accidents

## Abstract

**Background:**

The purpose of this study was to gain knowledge about the circumstances related to suicide among children and adolescents 15 years and younger.

**Methods:**

We conducted a psychological autopsy, collecting information from parents, hospital records and police reports on persons below the age of 16 who had committed suicide in Norway during a 12-year period (1993-2004) (n = 41). Those who committed suicide were compared with children and adolescents who were killed in accidents during the same time period (n = 43) and with a community sample. **Results: **Among the suicides 25% met the criteria for a psychiatric diagnosis and 30% had depressive symptoms at the time of death. Furthermore, 60% of the parents of the suicide victims reported the child experienced some kind of stressful conflict prior to death, whereas only 12% of the parents of the accident victims reported such conflicts.

**Conclusion:**

One in four suicide victims fulfilled the criteria for a psychiatric diagnosis. The level of sub-threshold depression and of stressful conflict experienced by youths who committed suicide did not appear to differ substantially from that of their peers, and therefore did not raise sufficient concern for referral to professional help.

## Background

The suicide rate for 10-14-year-olds in Norway has decreased from 1991-1995 (2.6/100,000) to 2006-2009 (0.7/100,000). In comparison, the suicide rate among children aged 10-14 in the United States was 1.6/100,000 in 1994, decreasing to 1.3/100,000 in 2004, but still representing the third leading cause of death in children younger than 14 [1,2]. The suicide rate for 15-19-year-olds in Norway has likewise decreased from 12.0/100,000 in 1991-1995 to 8.7/100,000 in 2006-2009. In the United Kingdom suicide rates for 10-19-year-olds decreased 28% between 1997 and 2003 [3].

There have been several studies of suicide in children and adolescents, but few specifically address the youngest age group [1,4-7]. Psychiatric illness has been considered the most important risk factor for adolescent suicide [6-8]. However, some studies of suicide victims younger than 15 years indicate that this youngest group may have less obvious suicidal intent and a lower frequency of psychiatric disorders [4,6]. Brent [4] compared suicide victims 16 years and younger (n = 35) with a mean age of 14.6, to those older than 16 (n = 105) in his psychological autopsy. He found mood disorder in 43% and any psychiatric disorder in 60% in the youngest group. Shaffer [9], in his psychological autopsy, found 57% with mood disorder among suicide victims under the age of 17, based on best-estimate diagnosis, but those who did not meet the criteria for any diagnosis had a significantly lower mean age (14.6). Shaffer, Groholt and Beautrais have all performed studies based on under 15-year-olds. Groholt [6] and Beautrais [8] found the prevalence of psychiatric disorder to be 43% and 23% respectively, while Shaffer [7] found that 30% were referred to psychiatric treatment. However, these studies were not based on interviews with parents.

It is uncertain whether a child understands the finality of death, and there are few studies on the subject [10-13]. A general consensus is that most children understand the irrevocable result of suicide by the age of 10 [14]. In order to improve our preventive work we need to know if children and adolescents who commit suicide exhibit common characteristics that could be used to recognize those at increased risk. Was the presence of a psychiatric disorder the main risk factor, as in older age groups? Which other factors contributed to the child's decision to commit suicide? To elicit answers to these questions, we performed a retrospective study, conducted as a psychological autopsy. All children and adolescents younger than 16 years who had committed suicide in Norway in the period between 1993 and 2004 were included. These children and adolescents were compared to children and adolescents who died in accidents during the same period. This comparison group was chosen because these children also suffered an unexpected death and died within the same time span. Finally, the results were compared with an age and gender matched community sample, intended to represent the average child.

Thus, the aim of the present study was 1. To shortly describe the suicides, 2. To assess psychopathology among children and adolescents who had committed suicide, compared to children and adolescents who died in accidents and children and adolescents in the community, 3. To assess characteristics among the suicide victims regarding socio-demographic factors and possible stressful conflicts prior to suicide.

## Methods

### Suicides

From Statistics Norway we received information about each of the 91 Norwegian residents 15 years and younger whose deaths were classified as suicide in the 12-year-period 1993-2004. Norwegian statistics used the International Classification of Diseases, 9^th ^revision (ICD-9) from 1993-1995 and the International Classification of Diseases, 10^th ^revision (ICD-10) from 1996-2004. The parents of the children received a letter inviting them to participate in the study. If the two parents had different addresses the invitation was sent to the mother only. A reminder letter was sent 3-4 weeks later to those who had not responded. Of the 91 parents, 43 (47%) agreed to participate, however, one later withdrew consent. Another respondent cancelled the interview appointment several times and was excluded, resulting in a total study population of 41 parents (45%), of which 16 (39%) had 15-year-old adolescents. All of the families had traditional Norwegian family names, indicating Norwegian ethnicity. The Regional Committee for Medical and Health Research Ethics gave us permission to contact the parents using only these two letters. In total, 48 parent pairs did not participate. The only information that we were able to obtain about their children was the age at death (mean age 13.5; range 9.10-15.11) and method of suicide (76% strangulation, 14% firearm, 4% intoxication, 2% drowning and 4% not specified). There were no significant differences between the participating and non-participating parents concerning the method of suicide or cause of death of their child. The parents, with two exceptions, had traditional Norwegian family names.

### Accidental deaths

We had to consider both historical bias and changes in youth culture from the period of study (1993-2004) to the period of data collection (2007-2009) when selecting a comparison group. If the child were still alive, the parent would probably not be able to recall what hers/his behavior was like at the age of 11, 12 or 14. However, if the child suffered a sudden and unexpected death at that very age, we assumed that the parent might recall the child's behavior in the same way as the parent of a child who had committed suicide: the parent would have the same historical bias as the parent in the suicide group. Therefore, we chose children and young adolescents who died between the ages of 10 and 15 in accidents between 1993 and 2004 in Norway, classified according to the ICD-9 (1993-1995) and ICD-10 (1996-2004), as our comparison group. Natural catastrophes and events in which the child was a passenger in a car, bus, train or boat were excluded to make the circumstances as similar to the suicides as possible: a single child's sudden death. Statistics Norway provided the parent's names and addresses in 106 cases nationwide, and we followed the same data collection procedure as for the suicide group. We were not able to locate three of the informants and seven were living in desolate places and were, for practical reasons, not contacted. Of the 96 we invited, 45 (47%) agreed to participate and all had traditional Norwegian family names. Among these 45 respondents one moved abroad before the interview was conducted and another case turned out to be a homicide and was excluded from the data analysis. Of the children whose parents chose not to participate the mean age was 13.2 years (range 10-15), which was not significantly different from the mean age of the children of the non-participating parents in the suicide group. The causes of death among the children in the control group were traffic accidents in which the child was a pedestrian, bicycle rider, etc. (41%); drowning (13%), jumps/falls (7%), strangulations (5%), intoxications (4%), deaths due to firearms (2%) or not specified (28%). With three exceptions, the parents had traditional Norwegian family names.

Both groups of parents preferred that the interview be conducted at their home, but 39% of the suicide group and 40% of the accident group chose other locations. The interviews had a mean duration of 132 minutes (SD = 26, range 75-132). Interviews in the suicide group averaged 137 minutes in length, and interviews in the accident group averaged 137 minutes in length, and interviews in the accident group averaged 127 minutes. The mean time span from the child's death to the interview was 10 years (SD = 4)in the suicide group and 9 years (SD = 4) in the accident group. Participants in the suicide and accident groups were spread throughout the country, with 29 parents or parent pairs (34%) living in cities and 55 (66%) living in villages or sparsely populated areas.

### Community controls

The community controls were selected from participants in the Bergen Child Study (BCS), a longitudinal population-based study of children's mental health and development. The study was launched in 2002 when the parents and teachers of all children in grades 2-4 in all elementary schools in Bergen (n = 9,155) were asked to complete a questionnaire. The children were 7-10 years old at time of wave 1, and 7,007 parents participated. A second wave was conducted when the children were 11-13-years old and information about 5,781 children was obtained. Two years later, when they were 13-15 years old, the study obtained information about 1,721 children and adolescents, (for more information about the BCS and related publications, see www.uib.no/bib ).The Strengths and Difficulties Questionnaire (SDQ) was included in all three waves, along with questions about a wide range of health issues. The community sample lived mainly in urban neighborhoods. From the BCS database, we randomly selected 10 community controls for each child or adolescent in the suicide group, matched for age and gender (n = 410).

### Procedure, suicides and accidents

The parents, one or both, were interviewed from 2007-2009. An experienced clinician (the first author), who is a specialist in adult psychiatry and child and adolescent psychiatry, conducted all interviews, of which 51% were recorded on tape. All diagnoses were assigned in accordance with the Diagnostic and Statistical Manual of Mental Disorders, 4^th ^edition, (DSM-IV, American Psychiatric Association, 1994). The first author used the Kiddie-SADS (see measurements) diagnostic interview and all available information in each case to arrive at the best clinical estimate of the child's diagnosis or diagnoses. To test the inter-rater reliability of the psychiatric diagnoses assigned, another psychiatrist used the SPSS to randomly select and assess 20% of the Kiddie-SADS interviews (17 in total). If the selected interview was not recorded, the next interview on the list was chosen. An acceptable inter-rater reliability was obtained, with a kappa of 0.82.

### Procedure, community controls

The BCS data included the results of the SDQ, the educational level and the working situation of the parent with whom the child lived on a daily basis, whether the child had been bullied by other children, and whether the child had been in contact with school psychology services or Child and Adolescent Mental Health Services (CAMHS). Not all information was collected in all three waves of the BCS, but the comparison children were selected from all three waves. The *n *was thus necessarily reduced for some of the variables in Table 1. Information was typically obtained from the mother. When data were missing from the mother on a particular variable, we included reports from the father, if available.

**Table 1:** Characteristics of the informants in the suicide group, the accident group and the community controls.

	Suicide group (*n = 41*)		Accident group (*n = 43*)		Statistics Suicides vs. accidents			Community controls (*n = 410*)		Statistics Suicide vs. community controls		
					OR	95%CI	p	n	(%)	OR	95%CI	p
	n	(%)	n	(%)								
Informant							.37					
Mother	14	(34)	21	(49)								
Father	4	(10)	4	(9)								
Both	23	(56)	18	(42)								
Informant, mean age												
Mother	42	(SD 4.6)	42	(SD 6.5)			.85					
Father	44	(SD 6.4)	45	(SD 6.2)			.81					
Ethnicity, mother												
Norwegian	40	(98)	43	(100)			.48					
Other	1	(2)	0									
Ethnicity, father												
Norwegian	38	(93)	43	(100)			.11					
Other	3	(7)	0									
Parents living together								(*n = 233*)*				
Yes	29	(71)	26	(60.5)	1.0			187	(80)	1.0		
No	12	(29)	17	(39.5)	.6	.3-1.5	.37	46	(20)	1.6	.7-3.5	.21
Education, mother	(*n = 40*)		(*n = 42*)					(*n = 373*)*				
Elementary/secondary school	25	(63)	31	(74)	1.0			259	(69)	1.0		
University	15	(38)	11	(26)	1.7	.7-4.3	.34	114	(31)	1.3	.7-2.7	.37
Education, father	(*n = 39*)		(*n = 42*)					(*n = 339*)*				
Elementary/secondary school	21	(54)	28	(67)	1.0			226	(67)	1.0		
University	18	(46)	14	(33)	1.7	.7-4.2	.26	113	(33)	1.7	.9-3.3	.11
Occupational status, mother								(*n = 147*)*				
Fulltime/part time employee	33	(80)	25	(58)	1.0			140	(95)	1.0		
Unemployed, other	8	(20)	18	(42)	.3	.1-.8	**.03**	7	(5)	4.8	1.6-14	**<.01**
Occupational status, father								(*n = 104*)*				
Fulltime/part time employee	28	(68)	30	(72)	1.0			98	(94)	1.0		
Unemployed, other	13	(32)	12	(29)	1.1	.4-2.9	.81	6	(6)	7.6	2.6-21.8	**<.01**

The suicide group is compared separately with the accident group and the community controls.

*The lower n is due to the fact that different information was assessed in the three BCS waves, and our community controls were from all three waves

### Measurements

We got information from the parents about various socio-demographic factors, all valid at the time of the child's death: parents' ethnicity, highest level of education (elementary/secondary school, university), occupational status (fulltime/part-time employee, unemployed/other), whom the child lived together with (both parents or not), residence (rural or urban), lifetime change of residence (never, once, twice or more), lifetime change of school (never, once, twice or more). We used approved Norwegian translations and back-translations of the selected instruments. To obtain diagnostic information, we used a semi-structured diagnostic interview, the Schedule for Affective Disorders and Schizophrenia for School Aged Children (6-18 years): Present and Lifetime Version (Kiddie-SADS-PL) [15]. The screening part includes questions about somatic complaints, irritability, self harm and the use of alcohol or other drugs. Depressive symptoms were noted as sub-threshold depression, in consistence with the definition of the term given by Fergusson [16]. The Kiddie-SADS-PL includes questions about learning difficulties, and also records suspicion about that the child has experienced any kind of assault and suicide attempts. The question about suicidal thoughts could not be answered by the parent so we asked if the child or adolescent had taken specific interest in the subject of suicide ("suicide interest"); read articles, lyrics, seen movies or TV-programs on the topic, been listening to destructive music glorifying suicide, or if they had often mentioned suicide to the parents in different ways, wanted to discuss it, etc., and we asked about expressed suicidal ideation and suicide threats. We also asked whether the child had been open to talking to hers/his parents about life difficulties. Furthermore, the parents were asked to categorize the mental and somatic health of the child (good or some problems) and to name personality traits of the child. Personality traits were classified into four diverging categories by the first author (vulnerable - touchy, worried - anxious, self and socially confident, impulsive - temperamental), not mutually exclusive. We obtained information about possible contact with school psychology services or CAMHS, either as an inpatient or outpatient (n = 9, 11%). According to Norwegian law, we received written approval from both parents (n = 8) to obtain the clinical record in order to access additional information relevant to the diagnostic assessment. For unknown reasons, we did not receive two of the requested records from CAMHS. We also asked about losses experienced by the child or adolescent and affecting her/him emotionally (such as loss of important person, family member, pet, peer, etc.), whether the child had experienced a stressful conflict within two weeks prior to death (with peers, school, parents or police) and if she/he had been bullied during the last years. Immediately after the interview the interviewer assigned a score on the Children's Global Assessment scale (C-GAS) [17] for the lowest level of functioning during the child's last year. The parents completed the Strengths and Difficulties Questionnaire, parent version, (SDQ-Nor) [18] in order to supplement the diagnostic considerations. The SDQ has 25 items, covering four problem domains (emotional, conduct, hyperactivity and peer problems) and personal strengths (pro-social behavior). Higher scores indicate more problematic attributes. A sub-score is generated for each problem domain, and a total difficulties score is generated by adding the problem domain scores. The SDQ also includes an impact factor, which assesses the overall level of distress created by these difficulties. If the SDQ had one missing value within a subscale, we assigned it the middle value of the other four within the group. When more than one value was missing, the item was not analyzed.

We received the police reports in significantly more of the suicide cases (37 of 41, 90%) than the accident cases (29 of 43, 67%) (OR=.22, CI=.06-.75, p=.01). The missing police reports were either not traceable or had been accidentally destroyed. A medical autopsy had been conducted in 37 of 84 cases (60% of the suicides, 52% of the accidents, no significant difference between the groups), and the report was attached. The death certificate from the hospital/physician and, if performed, the autopsy-report, give the reason for death which the Statistics Norway uses in their statistics, without using additional information from police-reports.

### Ethics

All required permissions were obtained from the Norwegian Directorate for Health and Social Affairs, the Norwegian Social Science Data Services, the Director General of Public Prosecution, the Directory of Residents, and Statistics Norway. The Regional Committees for Medical and Health Research Ethics approved the study.

### Statistical analyses

All analyses were performed with SPSS version 16.0 (SPSS Inc., Chicago, IL, USA). To describe the characteristics of the informants, we used Pearson's chi-square or Fisher's exact test, when possible, for the categorical variables and the t-test for independent groups for the continuous variables. The significance level was set at p ≤ 0.05. The odds ratio is abbreviated OR and the confidence interval CI. The suicide group was compared to the accident group and to the community control group in separate bivariate analyses. The independent significant variables in Table 2 were entered into a logistic regression analysis with suicide/accident as the dependent variable, using the 'enter' method in order to explore their independent effects on suicide, stratified for age and gender.

**Table 2:** Demographic characteristics, somatic health, mental health and stressors in the suicide group and the accident group.

	Suicide *(n = 41)*		Accident *(n = 43)*		Statistics		
	n	(%)	n	(%)	OR	95% CI	*p*
***Demographics***							
Gender							
Girls	12	(29)	20	(46.5)	1.0		
Boys	29	(71)	23	(53.5)	2.1	.9-.5.2	.12
Birth weight (grams)	3559	(SD 653)	3551	(SD566)			.95
Birth length (cm)	50.3	(SD 2.5)	50.6	(SD 2.2)			.67
Mean age (range)	14.4	(11.7-15.9)	13.5	(10.5-15.9)			**<.01**
Girls	14.4	(11.7-15.9)	13.2	(10-15.9)			**.05**
Boys	14.4	(12-15.9)	13.8	(10-15.9)			.10
Residence							
Rural	30	(73)	25	(58)	1.0		
Urban	11	(27)	18	(42)	1.9	.8-4.9	.17
Change of residence							
Never	17	(41.5)	25	(58)	1.0	.22	
Once	17	(41.5)	15	(35)	1.7	.7-4.2	.28
Twice or more	7	(17)	3	(7)	3.4	.8-15.2	.10
Change of school							
Never	32	(78)	39	(91)	1.0	.26	
Once	8	(20)	3	(7)	3.3	.8-13.3	.10
Twice or more	1	(2)	1	(2)	1.2	.1-20.3	.89
Somatic health							
Good	37	(90)	42	(98)	1.0		
Some problems	4	(10)	1	(2)	4.5	.5-42.5	.19
***Mental health***							
Mental health							
Good	27	(66)	38	(88)	1.0		
Some problems	14	(34)	5	(12)	3.9	1.-12	**.02**
Psychiatric diagnosis	*(n = 40)*						
Affective disorder	2	(5)	0	(0)			
Anxiety disorder	1	(3)	1	(2)			
Asperger disorder	1	(3)	0	(0)			
ADHD	1	(3)	1	(2)			
ADHD + conduct disorder	1	(3)	1	(2)			
Affective + anxiety	1	(3)	0	(0)			
Affective + anxiety + Asperger	1	(3)	0	(0)			
No disorder	32	(80)	40	(90)	3.3	.8-13.6	.07
Sub threshold depression							
No	28	(70)	42	(98)	1.0		
Yes	12	(30)	1	(2)	18.	2.2-146.3	**<.01**
C-GAS							
Mean	72	(SD 14)	83	(SD 14)			
Range	36-95		33-98				**<.01**
Specific learning difficulties							
No	38	(93)	37	(86)	1.0		
Yes	3	(7)	6	(14)	2.0	.5-8.0	.48
Personality trait*							
Vulnerable	21	(51)	4	(9)	10.	3.0-33	**<.01**
Worried	7	(17)	1	(2)	8.	1.0-70	.06
Confident	4	(10)	15	(35)	5.	1.4-16	**<.01**
Impulsive	6	(15)	0	-		1.7-2.7	**.02**
Suicidal behavior							
Suicidal threats	4	(10)	0	-			**.05**
Suicide interest	19	(47.5)	1	(2)	38.	4.7-303	**<.01**
Suicide attempts	5	(12.5)	0	-	2.2	1.7-2.8	**.02**
***Received help***							
School psychology services							
No	34	(83)	35	(81)	1.0		
Yes	7	(17)	8	(19)	.9	.2-2.7	1.0
Child & Adolescent Psychiatry							
No	36	(88)	39	(91)	1.0		
Yes	5	(12)	4	(9)	1.3	.3-5.4	.74
***Stressors***							
Suspected assault	5	(12)	1	(2)	5.8	.7-52.2	.10
Bullied							
No	29	(71)	37	(86)	1.0		
Yes	12	(29)	6	(14)	2.5	.9-7.6	.11
Experience of loss							
No	21	(51)	35	(81)	1.0		
Yes	20	(49)	8	(19)	4.1	1.5 -11.0	**<.01**
Stressful conflict							
No	16	(40)	38	(88)	1.0		
Yes	24	(60)	5	(12)	11.4	3.6-35	**<.01**

Differences are given as Odds Ratio with 95% Confidence Interval.

*10 (24%) in the suicide group and 22 (51%) in the accident group were not described with any of the personality traits

## Results

### Characteristics of the interview and the informants in the suicide and accident groups

The characteristics of the informants were applicable at time of the child's death (Table 1). Only one significant difference was found between the suicides and accidents: more mothers of suicide victims were employed. Compared to the community controls, academics were slightly more often (but not significantly more) represented, and both parents were more often unemployed. To investigate if this was due to differences between urban and rural residence, we compared the occupational status of the parents in the suicide group living in an urban environment (mothers n = 11, fathers n = 11) with parents of the community controls (mothers n = 147, fathers n = 104). We found no difference between the mothers whereas the fathers in the suicide group were more often unemployed than the community sample fathers (p=<.01).

We had no information on ethnicity of the parents in the community sample.

### Cause of death and method of suicide

Strangulation was the cause of death in 66% of the suicides and 5% (2) of the accidents. Firearms were used in 24% of the suicides, while jumping from a high surface and drowning each accounted for 5% of the suicide deaths. In the accident group hit by a vehicle accounted for 53%, drowning for 18%, intoxication for 5%, fall from a high surface for 5%, death on a tram/train/subway for 7% and other (fire, explosion, etc. during play) for 7% of all deaths. None of the police reports demonstrated any uncertainty as to whether the death was an accident or a suicide.

### Characteristics of the suicide victims and controls

The main characteristics of the suicide and accident victims are shown in Table 2. There were no significant differences between the suicide and accident group regarding socio-demographic variables, with one exception; the accident victims were younger. Mean birth weight and length were, according to the parents, within normal ranges (Norwegian birth statistics, Statistics Norway) in both groups. As community controls were matched with the suicides on age and gender, these variables were identical in the two groups.

### Mental health and stressors

#### Psychiatric symptoms and disorders

Table 2 shows the diagnoses according to Kiddie-SADS and all other available information. The only significant difference between the two groups was related to depression: four (10%) of the suicide victims experienced depression, while none of the accident victims did (OR=.45, CI=.4-.6, p=.05). Five of the suicide victims had one single diagnosis, while three had two or three diagnoses as shown. Among the accident victims two had single diagnosis and one had co-morbidity. In addition one of the accidents had enuresis and one had mental retardation, both not recorded as psychiatric disorder in the present paper. There were no differences related to self-harm or any other psychiatric diagnosis. However, the suicide victims had non-significantly more often a psychiatric diagnosis (p=.07). When we looked more closely at the answers of single items in the Kiddie-SADS, sub-threshold depression was reported in 8 (20%) in the suicide group and 1 (2%) in the accident group, in addition to those who had a depressive disorder. Thus, 12 of the suicide victims had depressive symptoms; among these 4 had a full depression. Among these 12, 10 (83%) were described as somewhat unwilling to talk to their parents about difficulties, 5 (41%) often complained of headaches or stomachaches, and 4 (33%) had been more irritable than usual before their death.

#### Personality

The following two examples were representative of how parents described their child:

"Jane was a very clever girl, both in theoretical and in practical activities. She did not give in until she achieved whatever goal she had, setting high standards for herself. She was a cautious girl, helpful, kind and attentive, particularly towards weaker peers, and she had a keen sense of justice. It bothered us a little that she never wanted to talk to us about how she perceived her own life, being as vulnerable and touchy as she was. But well, reserved, that's how most youngsters are about themselves, we guessed."

Jane committed suicide by strangulation at 15 years and 5 months of age.

"John was an active boy who participated in all kinds of sports and he did very well in school. He had a great sense of humor, was creative and loved to perform and write poems. He was quick-tempered all right, but shortly after a quarrel he smiled and was again a mostly very happy and content boy. He was also caring, considerate and vulnerable."

John shot himself when he was 13 years and 2 months old.

The suicide victims were often described as vulnerable or touchy, as reflected in these two vignettes. They also differed from the accident victims in their attitude towards the parents, as they talked less openly to them about difficulties in their lives, whereas only 2% among the accident victims had this attitude (OR = 13.5, CI = 1.6-111.5, p=<.01).

#### Suicidal behavior

All suicidal behaviors were significantly more frequent in the suicide group compared to the accident group. Some of the other characteristics are presented in Table 2. In the suicide group, 12% of the children had been in contact with CAMHS (two received help at the time of death, and three had ended their contact). In both the accident group and in the community group (n = 142), 9% had contact with CAMHS, which is not significantly different from the suicide group. The use of alcohol and/or other substances was 7 (17%) in the suicide group and 4 (9%) in the accident group (OR = 1.3, CI=.1-22.7, p = 1.0). This did not alter when 15-year-olds were analyzed separately.

#### Stressors

The suicide victims had significantly more often experienced a painful loss or a stressful conflict prior to death, compared to the accident victims. However, neither assault nor bullying differed significantly (Table 2). Sub-threshold depression was found in 3 (60%) of the bullied girls and 4 (57%) of the bullied boys in the suicide group and 1 (33%) of the girls and none of the boys in the accident group. After controlling for sub-threshold depression, the association between bullying and suicide was no longer significant.

#### Multivariate analyses

The variables of mental health and life stressors found to be significantly associated with suicide at the univariate level (Table 2) were put into a multivariate logistic regression in order to evaluate their adjusted associations (Table 3). Experience of loss, suicide interest, stressful conflict and having sub threshold depression significantly increased the risk of committing suicide, after adjusting for age, gender and the other significant variables. The expected association between C-Gas and suicide was not confirmed.

**Table 3:** Multivariate logistic regression analyses investigating possible differences between the suicide group and the accident group on significant variables describing aspects of mental health and actual stressors.

	Unadjusted			Adjusted*		
	OR	95% CI	*p*	OR	95% CI	*p*
Mental health problems	3.9	1.3-12.2	**.02**	.9	.1-8.6	.89
Suicide interest	38.0	4.8-303.6	**<.01**	22.3	1.9-264.9	**.01**
Loss	4.2	1.6-11.1	**<.01**	6.0	1.1-31.8	**.04**
Stressful conflict	11.4	3.7-35.2	**<.01**	10.6	1.7-68.2	**.01**
Vulnerable	10.2	3.1-33.9	**<.01**	.4	.1-2.3	.31
Sub threshold depression	18.0	2.2-146.3	**<.01**	49.8	1.2-2008.5	**.04**
C-Gas	.9	.9-.98	**<.01**	1.0	.9-1.1	.59
Gender	.5	.2-1.2	.11	.4	.1-2.6	.33
Age	1.6	1.1-2.1	**<.01**	1.5	.9-2.7	.13

* adjusted for age, gender and the other significant unadjusted variables presented in column two.

#### The Strength and Difficulties questionnaire

A logistic regression analysis was conducted to compare the five domain SDQ-sub-scores, total difficulties scores and impact score for the suicide group versus the accident group and for the suicide group versus the community control group (Table 4). The suicide victims had higher scores on both emotional and conduct problems, and they showed less pro-social behavior than accident victims. They also had higher total difficulties scores, representing a greater impact on their daily life and function compared to the accident group. When the suicide-versus-accident analyses were controlled for age and gender, there were no longer significant differences between the groups. There were no differences in sub-scores, total difficulties scores or impact scores in analyses comparing suicide victims with community controls. Even the emotional scores, which include depressive symptoms, did not differ significantly (p=.09).

**Table 4:** Logistic regression analyses of differences in SDQ sub scores, total difficulties score and impact score in the suicide group compared with the accident group and the community sample

	Suicide group		Accident group		Unadjusted			Adjusted*			Community sample		Unadjusted**		
					OR	95% CI	*p*	OR	95% CI	*p*	Mean	SD	OR	95% CI	*p*
	**Mean **	**SD**	**Mean**	**SD**											
Emotional Sub-score	1.3	1.7	.62	.92	1.52	1.1-2.2	**.03**	1.50	.9-2.4	.09	1.0	1.4	1.1	.9-1.4	.29
Conduct Sub-score	1.1	1.6	.41	1.0	1.56	1.0-2.4	**.04**	1.73	.9-3.5	.12	1.3	1.2	1.1	.9-1.4	.42
Hyperactivity Sub-score	1.9	2.2	1.9	2.0	.99	.8-1.2	1.0	.70	.5-1.0	.06	2.3	1.9	.91	.8-1.1	.29
Peer problems Sub-score	1.8	1.6	1.3	1.8	1.20	.9-1.6	.15	1.03	.7-1.4	.84	2.5	2.0	1.1	.9-1.4	.06
Prosocial Sub-score	1.1	1.3	.36	1.3	.71	.5-.9	**.04**	.88	.6-1.3	.48	1.8	1.6	1.1	.9-1.5	.15
Total difficulties score	6.3	4.7	4.3	3.9	1.12	1.0-1.3	**.05**	1.11	.9-1.2	.14	5.7	4.8	1.0	.9-1.1	.41
Impact score*** Yes	(28%)		(9%)		3.8	1.1-13.3	**.03**	.09	.1-6.1	.91	(21%)		1.5	.7-3.2	.29

*Adjusted for gender, age and the other sub scores.

**The Community control group was not analyzed adjusted because there were no unadjusted significant differences between suicide and community control group which on selection was matched for gender and age.

*** The impact score was answered yes (the behavior had impact on daily functioning) or no.

Using the Norwegian cut-off points between low and borderline risk of problems based on the parents' SDQ sub-scores [19], the percentage of children with a high emotional sub-score was significantly greater in the suicide group than in the accident group (p=.03). There were no significant differences between the suicide group and the accident or the community groups regarding the percentage with a score above the cutoff on any of the other variables. We also analyzed each SDQ item separately and the items with significant differences between the suicide group and the control groups are presented in Table 5. Multivariate logistic regression analyses were performed to assess the impact of the significant SDQ single items. The items 13 (often unhappy) and 22 (steals) contributed significantly to suicide risk, even when controlling for age and gender, whereas item 15 (easily distracted) seemed to reduce suicide risk.

**Table 5:** Individual SDQ items with significant differences between the suicide group and the accident group, and the suicide group and the community controls, respectively.

		Suicide group (*n = 39*)		vs. Accident group *(n = 43)*			vs. Community controls (*n = 410)*		
SDQ Single items		*Mean*	*SD*	*Mean*	*SD*	*p*	*Mean*	*SD*	*p*
SDQ-5	Often loses temper	.23	.54	.00	.00	<**.01**	.25	.53	.82
SDQ-6	Rather Solitary	.54	.79	.28	.55	.09	.33	.56	**.04**
SDQ-8	Many worries	.46	.64	.14	.41	**.01**	.23	.49	<**.01**
SDQ-9	Helpful if someone is hurt	1.62	.54	1.86	.47	.03*	1.70	.49	.31
SDQ-13	Often unhappy or depressed	.49	.76	.09	.37	<**.01**	.15	.39	**<.01**
SDQ-15	Easily distracted	.31	.66	.26	.58	.71	.57	.68	.02*
SDQ-19	Picked on or bullied	.36	.63	.16	.49	.12	.16	.45	**.01**
SDQ-22	Steals	.26	.55	.12	.39	.18	.03	.21	**<.01**
									

*The suicide group was less helpful and less distracted

#### Comparison with the community controls

For some variables, including contact with school psychology services, CAMHS and bullying, we were able to compare the suicide group to both the accident group and to the community controls. Contact with school psychology services in the suicide group was 17%, whereas among the community controls, only 6% had such contact (OR = 3.4, CI = 1.1-10, p=.05). Of the community controls 8% had been in contact with CAMHS, which was not significantly different from the suicide group (p=.36). In the suicide group 29% had been bullied, which was significantly more than the 9% of the community controls (OR = 4.0, CI = 1.7-9.1, p=<.01), but not significantly higher than the accident group (14%, p=.11).

## Discussion

The parents of suicide victims 15 years and younger described suicide interest, loss and sub threshold depression, not recognized as such at the actual time, and not causing much alarm. Therefore, according to the parents' perception, the majority appeared not to differ much from their peers. Even so, the children or adolescents often experienced a stressful conflict to which they were unable to immediately find good alternative solutions and ultimately ended up killing themselves. Thus, in spite of depressive symptoms, general vulnerability and stressful conflicts, these children and adolescents were not easily recognized as suicidal by their caregivers. In our suicide group we found, like previous studies [6,20,21], an overrepresentation of boys. The most common method of suicide in this young age group was strangulation, followed by the use of firearms, also this in line with other studies [8,21,22]. In the USA suffocation began occurring with increasingly frequency relative to firearms among 10-14-year-olds in the early 1990s [23].

### Mental Health

We found that 25% had a psychiatric diagnosis in our study, and approximately 30% had reduced mental health. This is in accordance with Shaffer's [7], Beautrais' [8] and Groholt's [6] findings of 13, 23 and 43%, respectively, among under-15-year-olds. None of these studies were based on interviews with parents, however. The findings are lower than in Brent's [4] and Shaffer's [9] psychological autopsy studies. The longer time-span from death to the interview may explain some of the differences. Also a lower number of informants in our study may be of importance. Further, the diagnostic interview used in this study may be stricter as a diagnostic tool. Still, the trend is the same: it seems the younger age group committing suicide has less psychiatric disorders than older age groups.

Orvaschel [24] tested the accuracy of retrospective diagnoses, although not in an autopsy setting, and concluded that such data are more likely to underreport past pathology and that false positives are not a major concern. Kelly [25] found evidence for the validity of psychological autopsy as a method to determine psychiatric diagnoses. The greater prevalence of sub-threshold depression in the suicide group confirms that depressed feelings are a risk factor for suicide also in this age group [6,9]. However, we should note that the diagnostic criteria for depression were often not met. This finding may explain the low rate of contact with CAMH, which is similar to Groholt findings [6].

In the suicide group, 12% had previously attempted suicide, based on the parents' knowledge. Brent [4] found 35% of the same age group, whereas Groholt [6] found zero. The number of unknown attempts may well be higher, but the rates of suicide attempts in younger age groups are expected to be lower than in older children. Though a suicide attempt is an important risk factor, its rare occurrence encourages us to look for other warning signs.

### Stressors

Stressful conflicts which were not considered important at the time they occurred were emphasized in retrospect. This is in agreement with Beautrais [8] who also found that arguments prior to suicide appeared to be about relative minor issues. Groholt [6] concluded that children younger than 15 suffered less mental health problems and fewer stressors compared to those older than 15, but that when present such risk factors affect the younger children in the same way. This is in agreement with our findings. When we examined the parents' description of the stressful conflict (caught stealing, having done some or something wrong, loss of dignity, experiencing hurtful comments, etc.), emotions like shame and/or guilt were likely to be present. Shame is the feeling a person experiences in response to failure in a social role and negative reactions from others that causes the person to evaluate his or her actions and conclude that he or she has done wrong [26]. Shame is, in general, a hard feeling to deal with and is often related to poor psychological well-being [27]. In combination with the personality traits we found in our sample, i.e. vulnerability and impulsiveness, it may prove dangerous. Shame is a feeling the individual tries to handle, using various strategies, and we found that the suicide victims exhibited specific ways of behavior when confronted with stressful conflicts, including avoidance of discussion of problems with parents, somatic complaints and irritability. This may mirror their developmental immaturity and in consequence restrict their ability to identify alternative solutions to problematic circumstances, as described by Pfeffer [14]. In this perspective, suicide may represent their attempt to cope with, or at least get away from, a situation perceived as unbearable.

The answers on SDQ item 22 indicate that the suicide victims were more often caught stealing than the average child. We found no studies confirming theft as a risk factor for suicide in this age group, but Shaffer [7] found that the most common situation before suicide was one in which the child knew that the parents were going to be told of some type of anti-social behavior that he or she had committed. We hypothesize that it is likely the parents of children who committed suicide were more likely to remember conflicts, to help them understand or give some kind of explanation for the suicide.

Half of the suicide victims had experienced some kind of loss that had affected them emotionally. Gutierrez [28] suggested that exposure to attempted suicide, completed suicide and non-suicidal death may influence attitudes toward life and death and, in turn, influence later suicidal behavior. Our study cannot answer the question of how such experiences may have affected their attitudes toward life and death. This is an interesting issue in need of further research.

Bullying is a well-known problem among children and one-third of the children who committed suicide had been bullied. In 2009, Klomek [29] studied exposure to bullying at age 8 and assessed suicide attempts or suicides later in life. He found that when boys were bullied they had an increased risk of suicide later in life if, combined with the presence of depression or conduct disorder. For girls, the risk of suicide increased independent of psychopathology. Of the 29% of children in our study who committed suicide and had been bullied, 57% of the girls and 60% of the boys had depressive symptoms at time of death. It is uncertain what came first, the depressive symptoms or the bullying, but the co-existence is often seen and can be mutually reinforcing, as Klomek describes [30] in her study of 13-19-year-old students. Bullying should be a major concern and a warning sign.

The fathers in the suicide group who lived in an urban environment were more often unemployed than the fathers in the community sample. We had no information as to whether these fathers were seeking further education or of possible psychopathology, which could have helped to explain the unemployment.

### Strengths and limitations

#### - Of the psychological autopsy as a method

The retrospective method of psychological autopsy provides information about suicide completers from a secondhand perspective. It involves the assessment of known risk factors, described by those who knew the deceased; in our study, the parents served as the informants. One must take into consideration that denial and forgetting are inherent to all psychological autopsy studies, as we tend to remember the deceased positively. On the other hand, a post-mortem label of depression and vulnerability may help the family explain why their child committed suicide. Information from official reports does, in some ways, serve as a correction. When both victims of suicides and accidents have "better" results on tested variables than the community controls, it reinforces the idea that problems are underreported when assessed retrospectively.

#### - Of this study

Suicide among younger children and adolescents is a rare event; thus, we chose a period of 12 years to obtain a somewhat satisfactory sample size. Brent and Shaffer both achieved roughly 70% participation rate in their studies [9,31]; our response rate was 45% which is low. The low sample size was a pervasive problem in making generalizations and drawing conclusions from the findings of our study. It also represents an increased possibility of overlooking real differences, i.e. committing type 2 errors.

However, this study is, to our knowledge, the first psychological autopsy study comparing children in this age group who committed suicide with children who died in accidents and a representative community sample. The mean time interval of ten years between the child's death and data collection represents a considerable source of error. However, the accident group had experienced the same time interval and would be subject to the same bias. Thus, when group differences were found, they were likely to represent real differences. The community controls should ideally have been subjected to the same time span before reporting, but this was not feasible. Whereas children in both the suicide and accident group lived in both urban and rural environments, the community sample was mainly urban.

The question of mislabeling, i.e. the classification of accidents as suicides or the opposite, is a possibility in all suicide research, and more so in the younger age group. However, in order to facilitate comparison with previous studies, we have used the official statistics.

Status as an ethnic minority is a known source of adjustment difficulties and an expected contributing factor to suicide. However, this group was not represented in our study. The findings of our study are probably more representative of an ethnically homogeneous group. The probable overrepresentation of educated parents should be kept in mind, making our sample biased towards resourceful ethnic Norwegian parents who would choose to participate in this kind of study. We were unable to explain the increased unemployment rates of the fathers in the suicide group. We did not obtain information about the mental health of the parents. This would have added important knowledge about bio-psycho-social factors known to influence the upbringing, living conditions and mental health of children and adolescents.

#### Ethical considerations

Inviting parents to talk about their deceased child is an emotionally stressful situation and should be thoroughly assessed. Bestows [32] examined the ethical concerns of psychological autopsies. He found that the interview was a positive experience for 48%, neutral for 39%, possibly negative for 5%, negative for 5% and impossible to assess for 3% of the participants. We did not systematically note the participants' immediate reaction to participation in the study.

## Conclusions

The psychiatric symptoms, mainly sub-threshold depression, the loss, the suicidal interest and actual stressful conflict that suicide victims aged 15 years and younger experienced, did not elicit enough worry among the caregivers to engage professional help at the actual time. The majority of them appeared not to differ much from their peers.

To the child and adolescent, the stressful conflicts he/she experienced prior to death may have been perceived as more important, shameful and difficult to handle than would be perceived by an adult. The caregivers of vulnerable children may be more reluctant to get involved in their child's problems than the caregivers of confident children. Maybe vulnerable children may benefit from a more proactive response from their caregivers.

## Competing interests

The authors declare that they have no competing interests.

## Authors' contributions

BG and AF contributed to the conceptualization of the study, were involved in the data analysis and contributed to the writing of the manuscript. AF performed the data collection and drafted the manuscript. AJL contributed with data from the BCS and to the writing of the manuscript. EK contributed to the writing of the manuscript. All authors read and approved the final manuscript.

## References

[B1] Dervic K Brent DA Oquendo MA Completed suicide in childhood Psychiatr Clin North Am 2008 31 271 291 10.1016/j.psc.2008.01.006 18439449

[B2] Kloos AL Collins R Weller RA Weller EB Suicide in preadolescents: who is at risk? Curr Psychiatry Rep 2007 9 89 93 10.1007/s11920-007-0076-9 17389116

[B3] Windfuhr K While D Hunt I Turnbull P Lowe R Burns J Suicide in juveniles and adolescents in the United Kingdom J Child Psychol Psychiatry 2008 49 1155 1165 10.1111/j.1469-7610.2008.01938.x 19017029

[B4] Brent DA Baugher M Bridge J Chen T Chiappetta L Age- and sex-related risk factors for adolescent suicide J Am Acad Child Adolesc Psychiatry 1999 38 1497 1505 10.1097/00004583-199912000-00010 10596249

[B5] Groholt B Ekeberg O Wichstrom L Haldorsen T Youth suicide in Norway, 1990-1992: a comparison between children and adolescents completing suicide and age- and gender-matched controls Suicide Life Threat Behav 1997 27 250 263 9357080

[B6] Groholt B Ekeberg O Wichstrom L Haldorsen T Suicide among children and younger and older adolescents in Norway: a comparative study J Am Acad Child Adolesc Psychiatry 1998 37 473 481 10.1097/00004583-199805000-00008 9585647

[B7] Shaffer D Suicide in childhood and early adolescence J Child Psychol Psychiatry 1974 15 275 291 10.1111/j.1469-7610.1974.tb01252.x 4459418

[B8] Beautrais AL Child and young adolescent suicide in New Zealand Aust N Z J Psychiatry 2001 35 647 653 10.1080/0004867010060514 11551281

[B9] Shaffer D Gould MS Fisher P Trautman P Moreau D Kleinman M Psychiatric diagnosis in child and adolescent suicide Arch Gen Psychiatry 1996 53 339 348 10.1001/archpsyc.1996.01830040075012 8634012

[B10] Melear JD Children's conceptions of death J Genet Psychol 1973 123 359 360 4764422 10.1080/00221325.1973.10532695

[B11] Mishara BL Conceptions of death and suicide in children ages 6-12 and their implications for suicide prevention Suicide Life Threat Behav 1999 29 105 118 10407964

[B12] Orbach I Glaubman H The concept of death and suicidal behavior in young children: three case studies J Am Acad Child Psychiatry 1979 18 668 678 10.1016/S0002-7138(09)62214-7 541472

[B13] Greydanus DE Calles J Jr Suicide in children and adolescents Prim Care 2007 34 259 273 10.1016/j.pop.2007.04.013 17666226

[B14] Pfeffer CR Childhood suicidal behavior. A developmental perspective Psychiatr Clin North Am 1997 20 551 562 10.1016/S0193-953X(05)70329-4 9323312

[B15] Kaufman J Birmaher B Brent D Rao U Flynn C Moreci P Schedule for Affective Disorders and Schizophrenia for School-Age Children-Present and Lifetime Version (K-SADS-PL): initial reliability and validity data J Am Acad Child Adolesc Psychiatry 1997 36 980 988 10.1097/00004583-199707000-00021 9204677

[B16] Fergusson DM Horwood LJ Ridder EM Beautrais AL Subthreshold depression in adolescence and mental health outcomes in adulthood Arch Gen Psychiatry 2005 62 66 72 10.1001/archpsyc.62.1.66 15630074

[B17] Shaffer D Gould MS Brasic J Ambrosini P Fisher P Bird H A children's global assessment scale (CGAS) Arch Gen Psychiatry 1983 40 1228 1231 10.1001/archpsyc.1983.01790100074010 6639293

[B18] Goodman R The Strengths and Difficulties Questionnaire: a research note J Child Psychol Psychiatry 1997 38 581 586 10.1111/j.1469-7610.1997.tb01545.x 9255702

[B19] Van RB Groholt B Heyerdahl S Clench-Aas J Self-reported strengths and difficulties in a large Norwegian population 10-19 years: age and gender specific results of the extended SDQ-questionnaire Eur Child Adolesc Psychiatry 2006 15 189 198 10.1007/s00787-005-0521-4 16724172

[B20] Dervic K Friedrich E Oquendo MA Voracek M Friedrich MH Sonneck G Suicide in Austrian children and young adolescents aged 14 and younger Eur Child Adolesc Psychiatry 2006 15 427 434 10.1007/s00787-006-0551-6 16685473

[B21] Shaw D Fernandes JR Rao C Suicide in children and adolescents: a 10-year retrospective review Am J Forensic Med Pathol 2005 26 309 315 10.1097/01.paf.0000188169.41158.58 16304461

[B22] Hoberman HM Garfinkel BD Completed suicide in children and adolescents J Am Acad Child Adolesc Psychiatry 1988 27 689 695 10.1097/00004583-198811000-00004 3198553

[B23] Lubell KMSMHCAE Methods of suicide among persons aged 10-19 years--United States, 1992-2001 MMWR Morb Mortal Wkly Rep 2004 53 471 474 15190241

[B24] Orvaschel H Puig-Antich J Chambers W Tabrizi MA Johnson R Retrospective assessment of prepubertal major depression with the Kiddie-SADS-e J Am Acad Child Psychiatry 1982 21 392 397 10.1016/S0002-7138(09)60944-4 7119313

[B25] Kelly TM Mann JJ Validity of DSM-III-R diagnosis by psychological autopsy: a comparison with clinician ante-mortem diagnosis Acta Psychiatr Scand 1996 94 337 343 10.1111/j.1600-0447.1996.tb09869.x 9124080

[B26] Lester D The role of shame in suicide Suicide Life Threat Behav 1997 27 352 361 9444730

[B27] Orth U Robins RW Soto CJ Tracking the trajectory of shame, guilt, and pride across the life span J Pers Soc Psychol 2010 99 1061 1071 21114354 10.1037/a0021342

[B28] Gutierrez P King CA Ghaziuddin N Adolescent attitudes about death in relation to suicidality Suicide Life Threat Behav 1996 26 8 18 9173613

[B29] Klomek AB Sourander A Niemela S Kumpulainen K Piha J Tamminen T Childhood bullying behaviors as a risk for suicide attempts and completed suicides: a population-based birth cohort study J Am Acad Child Adolesc Psychiatry 2009 48 254 261 10.1097/CHI.0b013e318196b91f 19169159

[B30] Klomek AB Marrocco F Kleinman M Schonfeld IS Gould MS Peer victimization, depression, and suicidiality in adolescents Suicide Life Threat Behav 2008 38 166 180 10.1521/suli.2008.38.2.166 18444775

[B31] Brent DA Perper JA Moritz G Allman C Friend A Roth C Psychiatric risk factors for adolescent suicide: a case-control study J Am Acad Child Adolesc Psychiatry 1993 32 521 529 10.1097/00004583-199305000-00006 8496115

[B32] Beskow J Runeson B Asgard U Ethical aspects of psychological autopsy Acta Psychiatr Scand 1991 84 482 487 10.1111/j.1600-0447.1991.tb03181.x 1776502

