# Correction: Differences between children and adolescents who commit suicide and their peers: A psychological autopsy of suicide victims compared to accident victims and a community sample

**DOI:** 10.1186/1753-2000-7-18

**Published:** 2013-06-17

**Authors:** Anne Freuchen, Ellen Kjelsberg, Astri J Lundervold, Berit Grøholt

**Affiliations:** 1 Department of Psychiatry, Faculty of Medicine, University of Oslo, N-0316, Oslo, Norway; 2 Department of Child and Adolescent Mental Health, Sorlandet Hospital, N-4610, Kristiansand, Norway; 3 Centre for Forensic Psychiatry, Oslo University Hospital, N-0407, Oslo, Norway; 4 Department of Biological and Medical Psychology, Uni Research, K.G. Jebsen Centre for Research on Neuropsychiatric Disorders, University of Bergen, N-5009, Bergen, Norway; 5 Institute of clinical medicine, Faculty of medicine, University of Oslo, N-0361, Oslo, Norway

## Correction

Since the publication of our article [1], we have noticed an error which occurs in three different places. We refer to 25% who met the criteria for a psychiatric diagnosis, but the correct value is 20%. Details of each correction are listed below.

Firstly, in the first sentence of the Results section of the Abstract, the statement “Among the suicides 25% met the criteria for a psychiatric diagnosis and 30% had depressive symptoms at the time of death”, should read “Among the suicides 20% met the criteria for a psychiatric diagnosis and 30% had depressive symptoms at the time of death”.

Secondly, in the first sentence of the Conclusions section of the Abstract, the statement “One in four suicide victims fulfilled the criteria for a psychiatric diagnosis”, should read “One in five suicide victims fulfilled the criteria for a psychiatric diagnosis”.

Finally, in the first sentence of the Discussion subsection entitled ‘Mental health’, the statement “We found that 25% had a psychiatric diagnosis in our study, and approximately 30% had reduced mental health”, should read “We found that 20% had a psychiatric diagnosis in our study, and approximately 30% had reduced mental health”.

Please note that the conclusions made in the original publication were based on the correct numbers as reported in the tables, and as such they are unaffected by the errors made.

We have also amended the affiliation for Astri J. Lundervold, which was incorrect in our original publication.
